# Book Reviews

**Published:** 1851-10

**Authors:** 


*A Practical Treatise on the Diseases and Injuries of the Urinary Bladder, the Prostate Gland, and the Urethra.* By S. D. Gross, M. D., Prof. of Surgery in the University of Louisville, etc., with one hundred and six illustrations. Philadelphia. Blanchard & Lea. 1851. 8 vo., pp. 726.

There are not many living medical writers on this continent to whom medical literature is under greater obligations than to Prof. Gross. There are but few members of the profession, at the present time, more competent to place the profession under fresh obligations than he. In the prime of life, and the full vigor of his intellectual faculties; with rich stores of practical experience and erudition, habits of industry, and a mind bent, not alone on the acquisition of knowledge, but its progress and diffusion, much as he has done already, it does not require prophetic skill to foresee that he will do much more, if life be spared. An earnest of this is furnished by the large and beautiful volume, the title-page of which prefixes this article. "The object of the work," quoting from the preface, "is to present in a systematic and connected form, a full and comprehensive account of the diseases and injuries of the urinary bladder, the prostate gland, and the urethra."

The author adds, "For many years I have myself felt the want of just such a book, or, at any rate, of one very much like it. No practitioner, who has been at all engaged in the active duties of his profession, can have failed to perceive, as well as to lament, the defects which exist in the literature of this particular department of the healing art. While every other organ of the body has had its expounder and monographist, it is a singular fact that no systematic treatise has yet appeared in the English language, on the maladies of the structures in question, especially those of the bladder, which are so common and so important, both in their pathological and practical relations. There has not yet been an attempt made on the part of any American writer to supply this deficiency; and it is no disparagement to the foreign works which have been republished in this country to assert, that, valuable as in many respects they are, they are far in arrear of the existing state of the science to which they relate." * * * * "It will thus be perceived that I have written the work, not merely for the sake of composing a book, but for the purpose of filling, if possible, a void in medical literature. I have endeavored to perform for the bladder, the prostate gland, and the urethra, what has been so well done by Lawrence, and Mackenzie for the eye, Hope for the heart, Budd for the liver, and Curling for the testis."

The following remark shows that Prof. G. is a lover of scientific toil. Few persons, we fancy, at the completion of so extensive a work, occupying three years in its preparation, will sympathize with him when he says, "The task, although not a light, has been an agreeable one. As the subject expanded under my eye, my feelings became more and more interested, and I almost regretted, when the last line was written, that my labor was ended."

Of the merits of the work as a contribution to surgical science, we shall not presume to speak. We leave this for those who devote attention to that province of medicine, and who are, in every way, more competent to offer a critical opinion. Measuring its value by our estimation of the attainments and ability of the author, we do not hesitate to express the belief that it is a contribution to our medical literature of which the profession of this country may well be proud.

---

*On Diseases of Menstruation, aud Ovarian Inflammation, in Connection with Sterility, Pelvic Tumors, and Affections of the Womb.* By Edward John Tilt, M. D., Physician to the Farringdon Gen. Dispensary, etc. New York. Samuel S., & Wm. Wood. 12 mo., pp. 286.

This work, which has been on our table for several weeks, has not been noticed on account of the gratification afforded by its perusal! In other words, we have been desirous of giving it an extended analytical review, instead of a brief notice, and having waited for leisure to accomplish this object, longer than our sense of propriety would dictate, we find ourself, at length, obliged to defer the undertaking until the appearance of a second edition, which, as we are informed by a note from the author, will be called for ere long. The fact just mentioned is the best evidence that the medical public can furnish, of the interest and value of the work. The subject opens a rich field for pathological and clinical researches — a field hitherto almost unexplored. The diseases of the ovaria have occupied a *terra incognita* in practical medicine. Here, as in other instances, advances in physiological knowledge precede developments in the study of morbid conditions. These organs have become invested with new interest and importance for the pathologist, by the discovery of relations which were not formerly suspected.

It is unnecessary to say that we refer to the new views respecting ovulation, and the connection which this process is supposed to sustain with the functions of the uterus.

In the work before us, Dr. Tilt presents the reader with the facts bearing on the diseases of the ovaria, which he has been able to gather from various sources, together with his own observations, which have been specially directed to this subject. As the work is small, requiring but little time for its perusal, and only a small pecuniary outlay, no practitioner who desires to keep pace with the progress of medical science should fail to purchase and and read it. For sale by Phinney & Co.

---

*Hints on the Profession of Medicine.* By Dr. Wm. Maxwell Wood, U. S. N.

The essay, with the above title, contained in the present and preceding numbers of this Journal, will, we presume, be perused by our readers with pleasure. It is a candid, clear, and cogent appeal, well calculated to correct popular errors, and remove prejudices, that it must be confessed, have obtained some currency by means of the diligent efforts of those whose selfish objects are promoted by diminishing public confidence in, and respect for the science and profession of medicine. Restricted to the pages of this Journal, however, the usefulness of the essay must be limited, inasmuch as it will not fall, to much extent, into the hands of the general reader. Believing that, if extensively circulated, it will do good, with the consent of the author, it will be issued speedily by G. H. Derby & Co., in a neat pamphlet form, and furnished to the profession at a cheap rate. This is done with the expectation that members of the Profession will take sufficient interest in the matter to purchase in quantities for distribution in the respective communities in which they reside. We beg leave to commend this course to those of our readers who are willing to do something to advance the character of the medical profession in public estimation. The purpose is one which affects, more or less, every practitioner. We are persuaded that if more pains were taken on the part of the profession to enlighten the public mind on subjects pertaining to medicine, much might be effected in the way of securing for it a more just appreciation, and interposing an obstacle to the progress of Quackery in its multifarious forms. It is because they are ignorant of the true character, scope, and means of progress belonging to legitimate medicine, that many persons are deluded by the specious promises, the sophistry, and the tricks of charlatanry. Is it not a duty which, as a profession, we owe to society, as well as ourselves, to endeavor to place the science which we love in a truer light before the public, and to secure a more intelligent, and consequently better appreciation of the art which we practice? These ends are to be attained, in part, by articles written for the popular reader, which shall set forth the truth plainly and clearly, relying upon arguments addressed to reason and common sense. Of such a character is the essay by Dr. Wood, and we trust that it will have an extensive circulation. As it will be stereotyped, copies will be furnished to any extent desired, by addressing the publishers, G. H. Derby & Co.

---

*Lecture on the Eruptive Fevers; as now in the course of delivery at St. Thomas's Hospital, London.* By George Gregory, M. D., Fellow of the Royal College of Physicians, of London, etc., etc. First American edition, with numerous additions and amendments by the author, comprising his latest views. With notes and an appendix embodying the most recent opinions on exanthematic pathology; and also statistical tables and colored plates. By H. D. Bulkley, M. D., Physician of the New York Hospital, etc., etc. New York: S. S. & W. Wood, Publishers, 261 Pearl Street. 1851. 8 vo., pp. 379.

The author of this work, Dr. Gregory, has had ample facilities for prosecuting the study of the Eruptive Fevers, more particularly variola, having been connected, for more than twenty years, with the London small-pox and vaccination hospital. The results of his experience are embodied in a short course of lectures annually delivered in the theatre of St. Thomas's Hospital. This work is a republication of these lectures as last delivered. The conversational style is preserved, which is often nearly as acceptable to the reader as to the listener. As a treatise on the department of practical medicine to which it relates, it cannot be said to have any very striking excellences, but it presents, clearly and concisely, the more important points embraced in the natural history, diagnosis, and treatment of the several exanthematæ. The additions and notes by Dr. Bulkley enhance the value of the work. Dr. B. is zealously devoted to the study of the *cutanei*, and is not only competent to supply deficiencies in the works of others, which relate to this province, but he would produce an excellent original treatise, were he to consent to undertake the task.

We should not omit to express our admiration of the fine typographical execution of this volume, as well as others emanating from the house of the worthy Publishers, the Woods, to whose extensive establishment we would commend our brethren who may visit New York.

